# *MSH3* modifies somatic instability and disease severity in Huntington’s and myotonic dystrophy type 1

**DOI:** 10.1093/brain/awz115

**Published:** 2019-06-19

**Authors:** Michael Flower, Vilija Lomeikaite, Marc Ciosi, Sarah Cumming, Fernando Morales, Kitty Lo, Davina Hensman Moss, Lesley Jones, Peter Holmans, Darren G Monckton, Sarah J Tabrizi, TRACK-HD Investigators, Peter Kraus, Rainer Hoffman, Alan Tobin, Beth Borowsky, S Keenan, Kathryn B Whitlock, Sarah Queller, Colin Campbell, Chiachi Wang, Doug Langbehn, Eric Axelson, Hans Johnson, Tanka Acharya, Dave M Cash, Chris Frost, Rebecca Jones, Caroline Jurgens, Ellen P ‘t Hart, Jeroen van der Grond, Marie-Noelle N Witjes- Ane, Raymund A C Roos, Eve M Dumas, Simon J A van den Bogaard, Cheryl Stopford, David Craufurd, Jenny Callaghan, Natalie Arran, Diana D Rosas, S Lee, W Monaco, Alison O’Regan, Cassie Milchman, E Frajman, Izelle Labuschagne, Julie Stout, Melissa Campbell, Sophie C Andrews, Natalie Bechtel, Ralf Reilmann, Stefan Bohlen, Chris Kennard, Claire Berna, Stephen Hicks, Alexandra Durr, C Pourchot, Eric Bardinet, Kevin Nigaud, Romain Valabre, ` gue, Stephane Lehericy, Cecilia Marelli, Celine Jauffret, Damian Justo, Blair Leavitt, Joji Decolongon, Aaron Sturrock, Alison Coleman, Rachelle Dar Santos, A Patel, Claire Gibbard, Daisy Whitehead, Ed Wild, Gail Owen, Helen Crawford, Ian Malone, Nayana Lahiri, Nick C Fox, Nicola Z Hobbs, Rachael I Scahill, Roger Ordidge, Tracey Pepple, Joy Read, Miranda J Say, Bernhard Landwehrmeyer, OPTIMISTIC Consortium, Ferroudja Daidj, Guillaume Bassez, Baptiste Lignier, Florence Couppey, Stéphanie Delmas, Jean-François Deux, Karolina Hankiewicz, Celine Dogan, Lisa Minier, Pascale Chevalier, Amira Hamadouche, Michael Catt, Vincent van Hees, Sharon Catt, Ameli Schwalber, Juliane Dittrich, Marie Kierkegaard, Stephan Wenninger, Benedikt Schoser, Angela Schüller, Kristina Stahl, Heike Künzel, Martin Wolff, Anna Jellinek, Cecilia Jimenez Moreno, Grainne Gorman, Hanns Lochmüller, Michael Trenell, Sandra van Laar, Libby Wood, Sophie Cassidy, Jane Newman, Sarah Charman, Renae Steffaneti, Louise Taylor, Allan Brownrigg, Sharon Day, Antonio Atalaia, Joost Raaphorst, Kees Okkersen, Baziel van Engelen, Stephanie Nikolaus, Yvonne Cornelissen, Marlies van Nimwegen, Daphne Maas, Ellen Klerks, Sacha Bouman, Hans Knoop, Linda Heskamp, Arend Heerschap, Ridho Rahmadi, Perry Groot, Tom Heskes, Katarzyna Kapusta, Jeffrey Glennon, Shaghayegh Abghari, Armaz Aschrafi, Geert Poelmans, Shaun Treweek, Fiona Hogarth, Roberta Littleford, Peter Donnan, Adrian Hapca, Michael Hannah, Emma McKenzie, Petra Rauchhaus, Sarah A Cumming, Darren G Monckton, Berit Adam, Catharina Faber, Ingemar Merkies

**Affiliations:** 1 Department of Neurodegenerative Disease and Dementia Research Institute, UCL, UK; 2 Institute of Molecular, Cell and Systems Biology, University of Glasgow, UK; 3 Instituto de Investigaciones en Salud (INISA), Universidad de Costa Rica, San José, Costa Rica; 4 School of Mathematics and Statistics, University of Sydney, Australia; 5 MRC Centre for Neuropsychiatric Genetics and Genomics, Cardiff University, UK

**Keywords:** Huntington’s disease, myotonic dystrophy, transcriptomics, movement disorders, association study

## Abstract

The mismatch repair gene *MSH3* has been implicated as a genetic modifier of the CAG·CTG repeat expansion disorders Huntington’s disease and myotonic dystrophy type 1. A recent Huntington’s disease genome-wide association study found rs557874766, an imputed single nucleotide polymorphism located within a polymorphic 9 bp tandem repeat in *MSH3*/*DHFR*, as the variant most significantly associated with progression in Huntington’s disease. Using Illumina sequencing in Huntington’s disease and myotonic dystrophy type 1 subjects, we show that rs557874766 is an alignment artefact, the minor allele for which corresponds to a three-repeat allele in *MSH3* exon 1 that is associated with a reduced rate of somatic CAG·CTG expansion (*P* = 0.004) and delayed disease onset (*P* = 0.003) in both Huntington’s disease and myotonic dystrophy type 1, and slower progression (*P* = 3.86 × 10^−7^) in Huntington’s disease. RNA-Seq of whole blood in the Huntington’s disease subjects found that repeat variants are associated with *MSH3* and *DHFR* expression. A transcriptome-wide association study in the Huntington’s disease cohort found increased *MSH3* and *DHFR* expression are associated with disease progression. These results suggest that variation in the *MSH3* exon 1 repeat region influences somatic expansion and disease phenotype in Huntington’s disease and myotonic dystrophy type 1, and suggests a common DNA repair mechanism operates in both repeat expansion diseases.

## Teaser

A genome-wide association study recently identified an *MSH3/DHFR* locus associated with Huntington’s disease progression. Flower, Lomeikaite *et al.* identify tandem repeat variants at this locus, and show that a three-repeat allele is associated with reduced somatic expansion, delayed onset and slower progression in Huntington’s disease and myotonic dystrophy type 1.

## Introduction

Huntington’s disease and myotonic dystrophy type 1 (DM1) are autosomal dominant disorders caused by CAG·CTG trinucleotide repeat expansions. Huntington’s disease is characterized by a progressive movement disorder, cognitive impairment and psychiatric symptoms (Bates *et al.*, 2014), and DM1 by myotonia, muscular dystrophy, cognitive impairment, cardiac conduction defects and endocrine dysfunction (Harper, 2001). No disease-modifying treatments are available for either (Bates *et al.*, 2015; Meola and Cardani, 2015).

Huntington’s disease is caused by a (CAG)n repeat expansion in *HTT* exon 1 and DM1 by a (CTG)n expansion in the 3′ untranslated region (UTR) of *DMPK* (Brook *et al.*, 1992; Bates *et al.*, 2014). In both, inherited repeat length is the major determinant of disease course, correlating inversely with the age at onset and positively with disease severity. The repeat is unstable, and expansion during germline transmission results in genetic anticipation (Hunter *et al.*, 1992; Bates *et al.*, 2014). Repeat tracts are also unstable in somatic cells, tending to expand over time, particularly in Huntington’s disease striatum (Kennedy *et al.*, 2003) and DM1 muscle (Ashizawa *et al.*, 1993), the most prominently affected tissues in each disease. Such expansion-biased, age-dependent and tissue-specific somatic instability is thought to contribute to disease onset and progression (Kennedy *et al.*, 2003; Shelbourne *et al.*, 2007; Swami *et al.*, 2009; Morales *et al.*, 2012).

In mouse models, the DNA mismatch repair proteins MSH2 and MSH3 are essential for CAG·CTG repeat expansion, and their inactivation limits expansion events and improves disease phenotype (van den Broek *et al.*, 2002; Foiry *et al.*, 2006; Dragileva *et al.*, 2009; Pinto *et al.*, 2013; Tome *et al.*, 2013). In patients with DM1, a candidate gene association study reported a coding single nucleotide polymorphism (SNP) (rs26279, p.A1045T) in *MSH3* exon 23 that was associated with the rate of somatic expansion (Morales *et al.*, 2016). Genome-wide association studies (GWAS) in patients with Huntington’s disease identified variation in DNA repair genes that modify disease course, and pathway analyses in each study further highlighted DNA repair (GeM-HD, 2015; Moss *et al.*, 2017; Lee *et al.*, 2017). Such variants also influence onset in other CAG expansion diseases, suggesting a common mechanism operates in conditions caused by repeat expansion (Bettencourt *et al.*, 2016). The lead variant in a recent GWAS linking *MSH3* with Huntington’s disease progression was the imputed SNP rs557874766, which nominally results in Pro67Ala at the N-terminus (Moss *et al.*, 2017).

However, rs557874766 is located within a 9 bp tandem repeat in exon 1 of *MSH3* and the 5′ UTR of the dihydrofolate reductase gene (*DHFR*) on the opposite strand. This repeat is polymorphic in copy number (Nakajima *et al.*, 1995; Morales *et al.*, 2016) and sequence (Morales, 2006), which led us to hypothesize that rs557874766 could be an alignment artefact. Additionally, the 500-bp region flanking the *MSH3* repeat is highly polymorphic, containing six SNPs and a 1-bp indel. We conducted targeted Illumina sequencing of the *MSH3* exon 1 region in 218 Huntington’s disease and 247 DM1 subjects, which allowed us to obtain accurate haplotype information for the region. Using whole blood RNA-Seq in Huntington’s disease, we investigated whether sequence variation at the *MSH3/DHFR* locus influences their expression.

## Materials and methods

### Cohorts

The 218 Huntington’s disease subjects were from TRACK-HD (Tabrizi *et al.*, 2009). The DM1_OPTIMISTIC_ cohort of 247 subjects was from OPTIMISTIC (van Engelen and Consortium, 2015) and the independent DM1_CostaRica_ cohort of 199 subjects was previously reported in Morales *et al.* (2016).

### Progenitor allele length

Progenitor pure CAG length for Huntington’s disease was determined by MiSeq sequencing (Ciosi *et al.*, 2018). Five subjects were excluded because they were part of a twin pair (*n* = 1) or the progenitor CAG length could not be unambiguously identified (*n* = 4) (Ciosi *et al.*, unpublished results). DM1 progenitor allele length was determined by small pool PCR (van Engelen and Consortium, 2015; Cumming *et al.*, in press). DM1 patients were tested for CCG repeat interruptions, known *cis*-modifiers of CTG repeat stability and disease phenotype (Cumming *et al.*, 2018, in press).

### Phenotypes

Two phenotypes were common to both cohorts: age at onset and rate of somatic expansion of the pathogenic CAG·CTG repeat. Huntington’s disease age at onset represents onset of motor symptoms (Tabrizi *et al.*, 2009). DM1 age at onset was subject self-assessment of the first occurrence of symptoms likely related to DM1 (Cumming *et al.*, in press). Somatic CAG·CTG expansion in blood was previously quantified in both cohorts (Ciosi *et al.*, unpublished results; Cumming *et al.*, in press). For Huntington’s disease MiSeq data, the measure of somatic expansion was the proportion of reads in the sample that correspond to somatic expansions (reads with more CAG repeats than the progenitor allele) relative to the number of reads obtained for the progenitor allele (Ciosi *et al.*, unpublished results). For DM1, it was the difference in number of repeats between the modal allele and the estimated progenitor allele length (Cumming *et al.*, 2018). In both cohorts, relative rate of somatic expansion corresponds to the variation in the measures of somatic expansion that is not explained by age and CAG·CTG repeat length. Positive values reflect a faster rate of somatic expansion.

Two phenotypes were only available for Huntington’s disease; progression score (Moss *et al.*, 2017) and gene expression. Progression score was derived for 213 TRACK-HD subjects in Ciosi *et al.* (unpublished results), as described in Moss *et al.* (2017). It measures typical Huntington’s disease progression that is not explained by age and pure CAG repeat length, with positive scores reflecting faster progression. Blood *MSH3* and *DHFR* expression levels were available for 108 Huntington’s disease subjects (Moss *et al.*, 2017).

### Illumina sequencing of *MSH3* exon 1

MiSeq amplicon sequencing, adapted from Ciosi *et al.* (2018), was used to genotype the *MSH3* exon 1 repeat and flanking variants (Supplementary Fig. 1). The region was amplified using locus-specific primers incorporating Illumina indexed adaptors (Supplementary Table 1) (Ciosi *et al.*, 2018). PCR was carried out using 10 ng of blood genomic DNA, 10% DMSO, 1 µM of each primer, 1× Custom PCR master mix (Thermo Scientific, SM0005), 0.048% (v/v) 2-mercaptoethanol and 0.5 U of Taq polymerase (Sigma) in a total volume of 10 µl. Thermal cycling conditions were: an initial denaturation at 96°C for 5 min, followed by 30 cycles of (96°C for 45 s), (60°C for 45 s) and (70°C for 2 min), with a final extension at 65°C for 1 min followed by 70°C for 10 min. Six hundred sequencing cycles were run 400 nt forward, 200 nt reverse. Quality control confirmed >80% of bases had Phred quality >30.

### Bioinformatic analyses

Genotyping was conducted on the University of Glasgow Galaxy platform (heighliner.cvr.gla.ac.uk). Paired-end reads were merged and aligned to multiple references corresponding to potential 9 bp repeat alleles (Supplementary material), followed by variant calling. For repeat homozygotes, haplotypes were confirmed from .sam files using Tablet (Milne *et al.*, 2013). The Galaxy workflow is available at https://www.myexperiment.org/workflows/5087.html. Conservation analysis used PhastCons and PhyloP (UCSC), with species sequence alignment in Clustal Omega.

### Transcriptome-wide association study

The transcriptome-wide association study (TWAS) method of Gusev *et al.* (2016) was used to impute cortical gene expression from 452 dorsolateral prefrontal cortex samples from the CommonMind Consortium (CMC, 2017) into the TRACK-HD GWAS of Huntington’s disease progression (*n* = 243) (Moss *et al.*, 2017). Following the Gusev *et al.* (2016) approach, we tested association between imputed cortical gene expression and Huntington’s disease progression.

### Statistical analyses

Linear regression modelling of genotype-phenotype correlation was conducted in R (R Core Team, 2013). An additive genetic model was used to score genotypes. For age at onset analysis, we controlled for CAG·CTG repeat length in Huntington’s disease and DM1, and for repeat interruptions in DM1 (Supplementary Table 4). Meta-analysis of somatic expansion and age at onset in Huntington’s disease and DM1 was conducted with METAL (Willer *et al.*, 2010). PLINK 1.07 (Purcell *et al.*, 2007) was used to derive allele frequencies, Hardy-Weinberg equilibrium (HWE) and linkage disequilibrium. Haplotype relationships were visualized as a network using median joining on NETWORK (Bandelt *et al.*, 1999).

### Data availability

Data are available from the corresponding author on request.

## Results

### Rs557874766 is an alignment artefact

**Figure 1:**
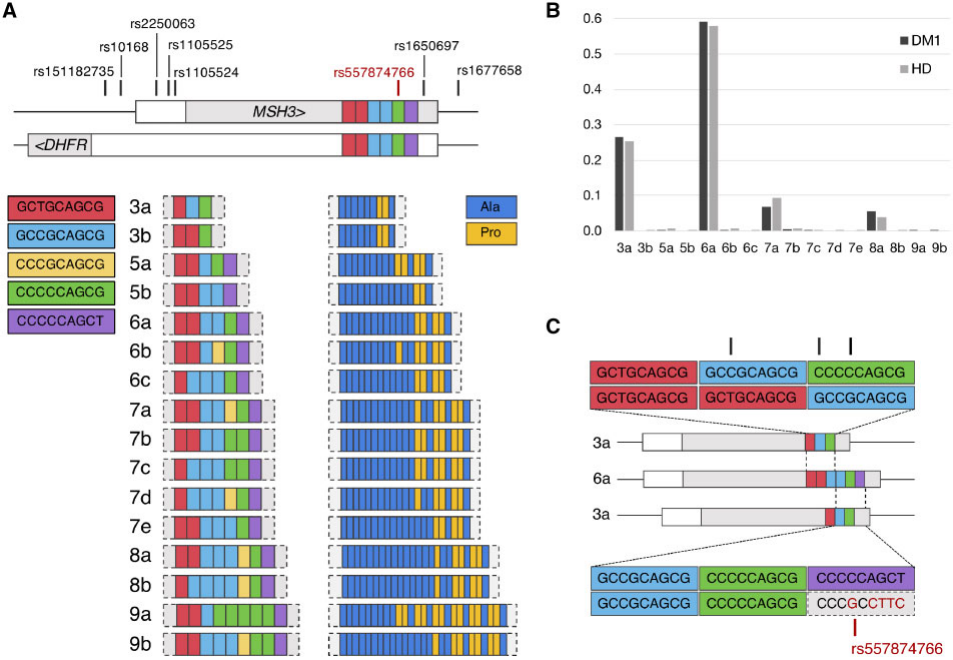
***MSH3/DHFR* 9bp tandem repeat allele structure and frequency observed in Huntington’s disease and DM1 cohorts.** (**A**) Schematic representation of the 9 bp tandem repeat alleles observed in this study and their coding potential. Repeat units are colour-coded by DNA and amino acid sequence. Location of the repeat and flanking variants in relation to *MSH3*/*DHFR* locus are shown in the *top* panel. This locus contains overlapping *MSH3* exon 1 and *DHFR* promoter regions. For both *MSH3* and *DHFR*, the 5’-untranslated region is shown in white and coding sequence in light grey. The direction of transcription is indicated by arrows for each gene. (**B**) Repeat allele frequencies observed in Huntington’s disease (HD) and DM1. Four common alleles, 3a, 6a, 7a and 8a, are observed in Huntington’s disease and DM1 cohorts at similar frequencies. (**C**) Schematic showing potential misalignments of 3a and 6a alleles, resulting in the apparent SNP rs557874766, shown in red on the lower alignment. Black marks in the *top* alignment represent mismatches that could be created in a similar manner as rs557874766, by misalignment of the 3a and 6a repeat alleles.

We observed 16 *MSH3* repeat alleles, differing in sequence and length from three to nine repeats (Fig. 1A and Supplementary Table 2). Alleles contained combinations of five types of repeat units, with coding potential for proline or alanine (Fig. 1A). They were numbered by repeat length, suffixed alphabetically by frequency i.e. ‘3a’ represents the most common three-repeat allele.

The most common allele in both cohorts, 6a (Fig. 1B), corresponds to the human reference sequence (NC_000005.10, GRCh38.p12). Illumina sequencing revealed that rs557874766 (Moss *et al.*, 2017) was not a SNP, but an alignment artefact resulting from the complex 9-bp repeat sequence (Fig. 1C). Individuals with the rs557874766 minor allele instead carry a three-repeat allele, 3a, the second most common allele observed in both cohorts. Two subjects with Huntington’s disease imputed as homozygous for the rs557874766 major allele were determined to be heterozygous for the 3a repeat allele by both Illumina and Sanger sequencing (Supplementary Fig. 2), highlighting the importance of directly genotyping such complex loci. We conclude that rs557874766 does not exist in the form of an SNP and results from incorrect alignment of the 3a allele to the reference 6a allele (Fig. 1C).

The *MSH3* exon 1 repeat region is poorly conserved between species, with mean scores of 0.29 [standard deviation (SD) 0.41] and 0.25 (SD 0.91) in PhastCons and PhyloP, respectively (Supplementary Table 3). Sequence alignment of 20 mammalian reference genomes showed most have two repeats (Supplementary Fig. 3). Together with a four- and a five-repeat allele, the 3a allele has been observed in gorillas and chimpanzees, suggesting 3a is an ancestral allele in humans (Morales, 2006).

### *MSH3/DHFR* variants are associated with rate of somatic expansion and disease phenotypes in Huntington’s disease and DM1

**Figure 2:**
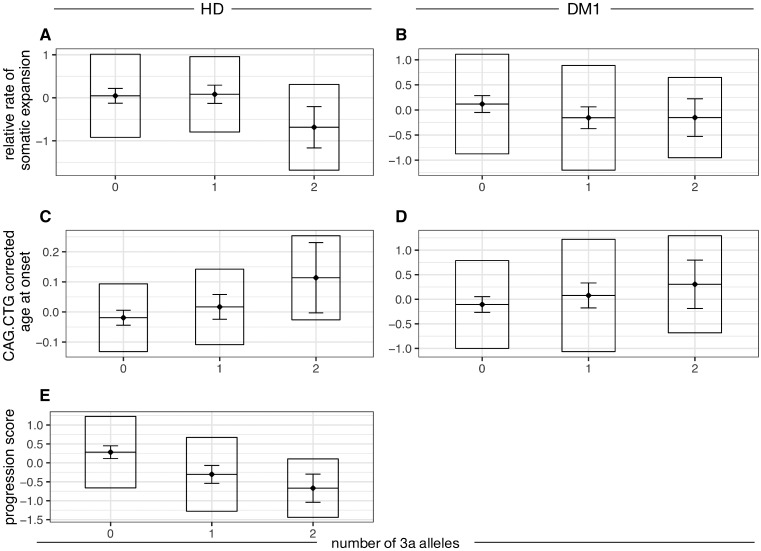
**The number of *MSH3* 3a repeat alleles is associated with Huntington’s disease and DM1 phenotypes.** Boxplots for three measures of disease phenotype are shown: rate of somatic expansion corrected for the inherited CAG·CTG length in Huntington’s disease (**A**) and for the inherited CAG·CTG length and variant repeats in DM1 (**B**); age at onset corrected for the inherited CAG·CTG length in Huntington’s disease (**C**) and DM1 (**D**); progression score in Huntington’s disease (**E**). For each dataset, the diamond and horizontal line spanning the diamond indicate the mean, the box the standard deviation and the whiskers the 95% confidence intervals of the mean. HD = Huntington’s disease.

The 3a allele correlated negatively with relative rate of somatic expansion in subjects with Huntington’s disease (*P* = 0.032) and showed similar effect direction, though above nominal significance, in DM1 (*P* = 0.053) (Fig. 2 and Supplementary Table 2). Additionally, 3a was associated with delayed age at onset by 1.05 years (*P* = 0.0029) and slower progression in Huntington’s disease by 0.52 units (*P* = 3.86 × 10^−7^), which corresponds to 0.37 and 0.10 units per year on the UHDRS total motor score and total functional capacity, respectively. In DM1, the association between 3a and age at onset showed a consistent effect direction, approaching significance (*P* = 0.061). In meta-analysis, 3a was significantly associated with relative rate of somatic expansion (*P* = 0.004) and age at onset (*P* = 0.003) in Huntington’s disease and DM1. Detailed analysis of the relationship between repeat alleles and phenotypes (Supplementary Table 5) shows that the 3a allele accounts for the reduced somatic expansion rate, delayed onset and slower progression observed in Huntington’s disease. The association with somatic expansion appears to be driven by 3a homozygotes, whereas that with progression seems to follow an additive pattern with the number of 3a alleles. For onset, the pattern of association is unclear. In DM1, the number of seven-repeat alleles was associated with reduced expansion rate (Supplementary Table 5).

**Figure 3:**
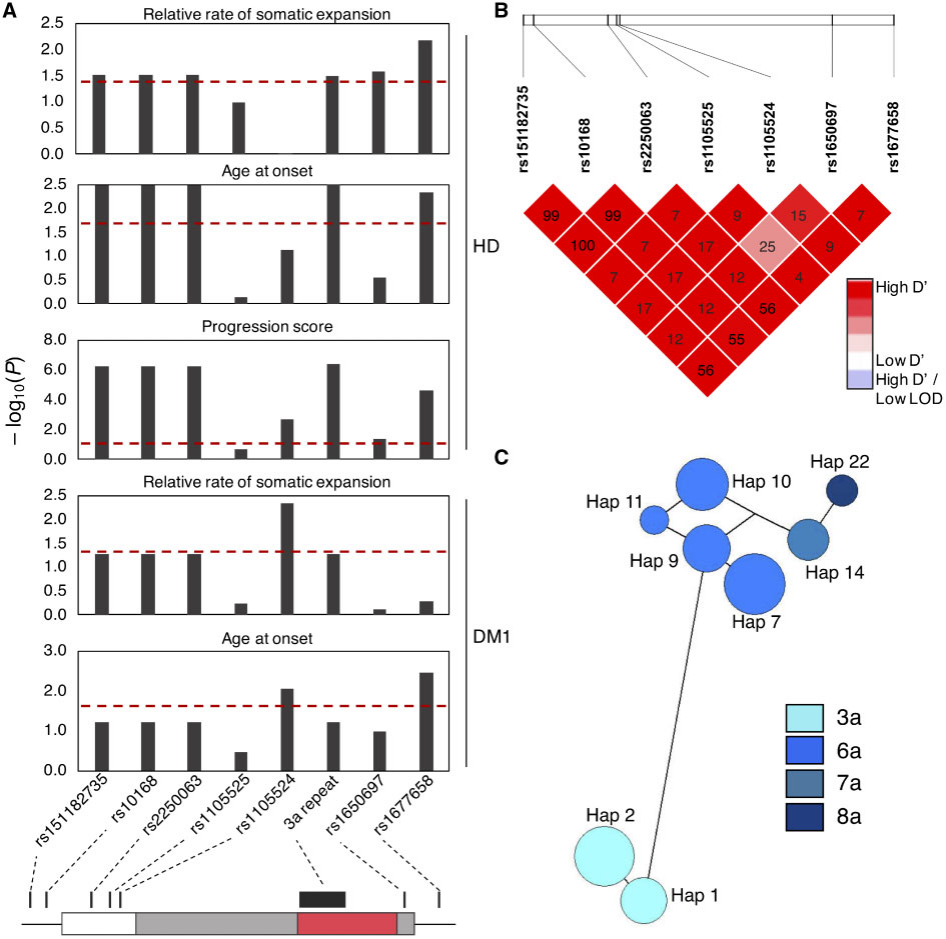
**Variants at the *MSH3*/*DHFR* locus are associated with phenotypes in Huntington’s disease and DM1.** (**A**) Bar charts showing associations between variant genotypes and disease phenotypes: relative rate of somatic expansion and age at onset corrected for the CAG·CTG length and progression score for Huntington’s disease, and rate of somatic expansion and age at onset corrected for the CAG·CTG length and repeat interruptions for DM1. Each bar represents association for a single variant. Red dotted line represents the *P* = 0.05 significance threshold. Variant location in relation to the *MSH3* exon 1 region is shown in the *bottom* panel. White box = 5’ untranslated region; grey = coding sequence; red = *MSH3* repeat region; intron is shown by a black line. (**B**) Linkage disequilibrium heat map for the seven variants flanking the *MSH3* repeat. Colour intensity represents the D’ value for each SNP pair. R^2^ values are indicated in text for each variant pair. (**C**) Haplotype network for eight haplotypes with frequency > 0.035 observed at the *MSH3* exon 1 region. Circles represent different haplotypes. The size of the circle is proportional to the number of individuals with a particular haplotype. Each haplotype is connected with the most similar haplotype by a line. Length of the line represents the number of genotypes that are different between each two haplotypes. Circles are colour coded according to the repeat allele found on the haplotype.

In addition to testing repeat allele effects, we also assessed correlation between flanking SNP genotypes and disease phenotypes. All the flanking variants were in HWE (Supplementary Table 6) and in strong linkage disequilibrium with each other (Fig. 3B). Three variants (rs151182735, rs10168 and rs2250063) were in nearly complete linkage disequilibrium with the 3a allele, and as such were as significantly associated with phenotypes (Fig. 3A and Supplementary Table 6). All three are non-coding variants 5’ to the repeat and their alternative alleles are associated with reduced *MSH3* and *DHFR* expression in the prefrontal cortex (CMC, 2017) and in multiple tissues in GTEx (GTEx, 2015) (Supplementary Table 7). Three SNPs, rs1105524, rs1650697 and rs1677658, also correlated with some phenotypes, though not uniformly (Fig. 3A and Supplementary Table 6). Rs1105524 and rs1677658 are non-coding variants, whereas rs1650697 corresponds to Ile79Val. All three are expression quantitative trait loci (eQTL) for *MSH3* and *DHFR* in the prefrontal cortex (CMC, 2017) and in multiple tissues in GTEx (Supplementary Table 7). Previously, in a separate DM1 cohort (DM1_CostaRica_), Morales *et al.* (2016) reported association between both rs1677658 (*P* = 0.009) and rs10168 (*P* = 0.031) and somatic expansion, though neither survived correction for multiple testing for the candidate SNPs analysed. However, the direction of effect for both SNPs was the same as in the present study, and a significant association in meta-analyses with the two DM1 cohorts (rs1677658 *P* = 0.03, rs10168 *P* = 0.004) and all three DM1 and Huntington’s disease cohorts (rs1677658 *P* = 8.85 × 10^−4^, rs10168 *P* = 3.37 × 10^−4^) suggests these variants influence somatic expansion (Supplementary Table 6). Morales *et al.* (2016) reported an association between somatic expansion and age at onset, though the direct effect of *MSH3* genotype on age at onset was not found to be significant. In the present study, meta-analyses of the two DM1 cohorts (rs1677658 *P* = 0.009, rs10168 *P* = 0.04) and all three DM1 and Huntington’s disease cohorts (rs1677658 *P* = 8 × 10^−4^, rs10168 *P* = 0.003) found the *MSH3* genotype was significantly associated with age at onset (Supplementary Table 6). Meta-analyses of the three-repeat allele with all three DM1 and Huntington’s disease cohorts provide further support for its protective effect on somatic expansion (DM1_OPTIMISTIC_ + DM1_CostaRica_*P* = 0.004, DM1_OPTIMISTIC_ + DM1_CostaRica_ + Huntington’s disease *P* = 3.46 × 10^−4^) and age at onset (DM1_OPTIMISTIC_ + DM1_CostaRica_*P* = 0.04, DM1_OPTIMISTIC_ + DM1_CostaRica_ + Huntington’s disease *P* = 0.003) (Supplementary Table 2).

The associations of SNPs with phenotypes were conditioned on the effects of *MSH3* repeat alleles (Supplementary Table 8). As rs151182735, rs10168 and rs2250063 perfectly correlated with 3a, their independent effects could not be determined (Supplementary Table 6). With the exception of rs1677658 (linkage disequilibrium with 3a: r^2 ^= 0.610) and rs1650697 (linkage disequilibrium with 3a: r^2 ^= 0.143), whose alternative alleles were associated with delayed and early age at onset, respectively in the combined Huntington’s disease and DM1 meta-analysis (*P* = 0.015 and *P* = 0.029; Supplementary Table 8), there was no significant evidence for association between SNPs and expansion rate, onset or progression independent of repeat alleles.

Considering variants with minor allele frequency >0.1 and all of the repeat alleles, we observed 25 haplotypes in the region, named Hap1 to Hap25 (Supplementary Table 9). The 3a repeat allele occurs on both Hap1 and Hap2, which differ only in the presence of the rs1677658 alternative allele on the more common Hap2. Hap1 was associated with reduced somatic expansion in DM1 (*P* = 0.032) and slower progression in Huntington’s disease (*P* = 0.020), whereas Hap2 was associated with reduced somatic expansion (*P* = 0.021) and delayed onset (*P* = 4.03 × 10^−5^) in both Huntington’s disease and DM1, and with slower progression (*P* = 1.64 × 10^−5^) and reduced expression of *MSH3* (*P* = 0.024) and *DHFR* (*P* = 1.12 × 10^−3^) in Huntington’s disease (Supplementary Table 9).

Overall, this analysis clarifies the sequence and variants present in *MSH3* exon 1 and demonstrates that *MSH3* repeat variants are associated with disease phenotypes in both Huntington’s disease and DM1.

### *MSH3* and *DHFR* expression in blood is associated with repeat alleles

**Figure 4:**
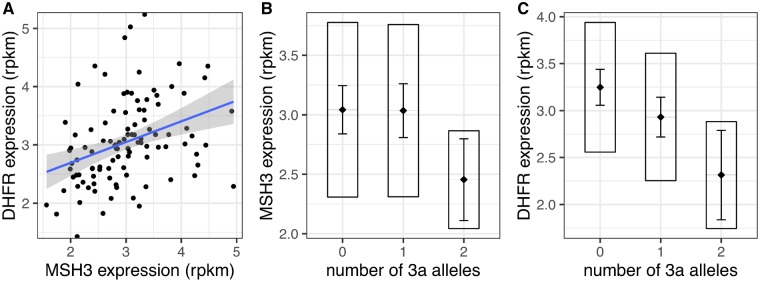
**Association of the *MSH3* 3a allele with *MSH3* and *DHFR* expression in Huntington’s disease whole blood.** Whole blood RNA-Seq in a subset of 108 Huntington’s disease subjects. (**A**) Significant correlation between *MSH3* and *DHFR* expression levels (r^2 ^= 0.120, *P* = 2.06 × 10^−4^). Grey area around the blue regression line represents 95% confidence interval of the model. (**B**) Homozygosity for *MSH3* 3a repeat allele is associated with lower *MSH3* expression in blood (*P* = 0.028). (**C**) *MSH3* 3a repeat allele is associated with lower *DHFR* expression (*P* = 2.33 × 10^−4^). Rpkm = reads per kilobase of transcript per million mapped reads. In boxplots, the diamond and horizontal line spanning the diamond indicate the mean, the box indicates the standard deviation and the whiskers indicate the 95% confidence intervals of the mean.

Each 3a allele was associated with reduced *DHFR* expression (*P* = 2.48 × 10^−4^; Fig. 4C) and homozygosity for 3a was associated with reduced *MSH3* expression (*P* = 0.0273; Fig. 4B), whereas each 7a or 8a allele was associated with increased *MSH3* expression (*P* = 8.55 × 10^−4^ and *P* = 8.26 × 10^−3^, respectively). The sum of *MSH3* repeat lengths on both alleles appeared to correlate with *MSH3* (*P* = 7.00 × 10^−3^) and *DHFR* expression (*P* = 1.76 × 10^−3^), which would suggest increasing repeat length increases expression of both (Supplementary Fig. 4). However, a more detailed analysis of *MSH3* repeat alleles (Supplementary Table 5) shows the number of seven- or eight-repeat alleles is associated with increased expression of *MSH3* (*P* = 4.53 × 10^−6^), and that this explains the apparent association with the sum of repeat lengths. In this relatively small cohort, *MSH3* (age at onset *P* = 0.446, progression *P* = 0.440) and *DHFR* (age at onset *P* = 0.911, progression *P* = 0.284) expression in blood were not themselves directly associated with disease phenotype. *MSH3* expression was not significantly associated with somatic expansion (*P* = 0.625), whereas the association of *DHFR* expression, while nominally significant (*P* = 0.049), did not survive correction for the number of phenotypes tested.

In the detailed analysis, the number of three-repeat alleles was associated with reduced *DHFR* expression (*P* = 2.33 × 10^−4^; Fig. 4C), and this was sufficient to explain the apparent association of *DHFR* expression with other repeat alleles (Supplementary Table 5), including that observed with increasing total repeat length. *DHFR* and *MSH3* expression are correlated (r^2 ^= 0.120, *P* = 2.06 × 10^−4^; Fig. 4A). However, association between *DHFR* and three-repeat alleles remains significant after correcting for *MSH3* expression (*P* = 7.51 × 10^−4^), and association between *MSH3* and seven- or eight-repeat alleles remains significant after correcting for *DHFR* expression (*P* = 1.30 × 10^−7^). In the best-fitting model for *DHFR* expression, the alternative allele at rs1105524 (linkage disequilibrium with 3a: r^2 ^= 0.192) increases and rs1650697 decreases *DHFR* expression independently of the three-repeat alleles (Supplementary Table 8). Otherwise, the repeat allele is the major determinant of *MSH3* and *DHFR* expression, and there is no evidence of independent SNP effects.

### *MSH3* expression in cortex is associated with onset and progression in Huntington’s disease

In a TWAS, increased expression of both *MSH3* and *DHFR* in prefrontal cortex (CMC, 2017) was associated with faster progression in TRACK-HD (Moss *et al.*, 2017) at similar levels of significance (*P* = 2.52 × 10^−6^ and *P* = 4.08 × 10^−6^, respectively; Supplementary Table 10), making it difficult to distinguish which is more functionally relevant. This ties in with the observation that SNPs significantly associated with somatic expansion, age at onset and progression (Supplementary Table 6) were eQTLs for both *MSH3* and *DHFR* in CMC data. Notably, however, increased *MSH3* expression was significantly associated with early onset (*P* = 1.71 × 10^−3^) in a TWAS of the GeM dataset (GeM-HD, 2015), while *DHFR* expression was not significantly associated with onset (Supplementary Table 10). This favours *MSH3* over *DHFR* expression as a modifier of Huntington’s disease course.

## Discussion

*MSH3* has recently been identified as a genetic modifier of somatic instability in DM1 (Morales *et al.*, 2016), and progression in Huntington’s disease (Moss *et al.*, 2017). The *MSH3* signal in the GWAS of Huntington’s disease progression was driven by an imputed SNP, rs557874766, located within a 9 bp tandem repeat sequence in exon 1 of *MSH3*, which is also in the 5′ UTR of *DHFR* on the opposite strand. *MSH3* and *DHFR* are organized head-to-head, transcribed in opposite directions and are regulated by the same promoter. Here we demonstrate that rs557874766 is an alignment artefact and corresponds to a three-repeat allele, 3a, which was the shortest repeat allele observed and is likely ancestral. At the protein level, *in silico* modelling predicts that 6a results in the gain of a surface α-helix (Kallberg *et al.*, 2012) at the N-terminus of MSH3.

A total of 16 *MSH3* repeat alleles were observed, varying in sequence and length from three to nine repeats. Repeat alleles 6a and 3a are the first and second most common in this European cohort, though previous studies suggest a seven-repeat allele may be second most common in East Asian populations (Nakajima *et al.*, 1995). In Huntington’s disease, 3a was associated with reduced somatic expansion, delayed onset and slower progression. In DM1, each 3a allele showed a trend towards reduced somatic expansion and delayed onset but was significantly associated with both measures in meta-analysis of Huntington’s disease and DM1. Longer seven-repeat alleles were associated with reduced somatic expansion only in DM1. Whether this reflects a subtle difference in MSH3 biology between the two disorders, or simply a sampling error, remains undetermined.

The *MSH3* repeat lies between binding domains for PCNA (Clark *et al.*, 2000) and EXO1 (Schmutte *et al.*, 2001), both of which are involved in mismatch repair (MMR) (Kleczkowska *et al.*, 2001). PCNA is a sliding clamp that participates in DNA replication, but in MMR it delivers MSH proteins to mismatches and increases binding specificity (Flores-Rozas *et al.*, 2000). EXO1 excises the daughter strand after mismatch recognition, as well as being involved in end resection during homologous recombination (Goellner *et al.*, 2015). The *MSH3* repeat region is poorly conserved between species, with other mammals having between zero and five repeats. This lack of evolutionary constraint suggests functional redundancy in the MMR pathway and a lack of a major effect of N-terminal MSH3 variation outside the context of repeat expansion disease. Unlike other MMR components, germline heterozygous *MSH3* mutations are not associated with increased risk of cancer, most likely because MSH2/MSH6 can also initiate repair at replication errors (Edelmann *et al.*, 2000; Jiricny, 2006; Haugen *et al.*, 2008).

Three non-coding variants 5’ of the repeat were in near complete linkage disequilibrium with 3a, so it is not possible to determine their independent effects on disease phenotypes. All three are associated with reduced *MSH3* expression in multiple tissues, including cortex (CMC and GTEx). Controlling for repeat alleles, no SNPs were significantly associated with phenotypes, except the intronic rs1677658 and the exon 1 rs1650697 variants, which contributed to delayed or early onset, respectively in the combined Huntington’s disease and DM1 dataset. Rs1677658 was associated with reduced *MSH3* and *DHFR* expression (CMC and GTEx), whereas rs1650697 was associated with increased *DHFR* in Huntington’s disease blood, as well as multiple tissues in GTEx. Hap2, the *MSH3* haplotype most significantly linked with reduced somatic expansion and delayed onset in Huntington’s disease and DM1, and with slower progression in Huntington’s disease, contains the 3a allele, along with alternative alleles of non-coding variants rs151182735, rs10168 and rs2250063, which are in complete linkage disequilibrium with it, and rs1677658. It is thus difficult to assess which (if any) *MSH3* variants (repeats or SNPs) are driving associations with disease phenotypes, and further investigation in a larger sample is warranted.

Whole blood transcriptomic analysis in a subset of the Huntington’s disease patients found the 3a allele was associated with reduced expression of *MSH3* and *DHFR*, and seven- or eight-repeat alleles with increased *MSH3* expression. *DHFR*, which shares a promoter with *MSH3* (Drummond, 1999), is a ubiquitously expressed enzyme involved in purine, thymidylic acid and amino acid synthesis, but has not previously been implicated in Huntington’s disease pathogenesis. Our TWAS found that increased expression of *MSH3* and *DHFR* in cortex are associated with faster Huntington’s disease progression (Moss *et al.*, 2017). While *MSH3* expression was significantly associated with early onset in our GeM TWAS (*P* = 1.71 × 10^−3^) (GeM-HD, 2015), *DHFR* expression was not associated with disease course. This is consistent with Huntington’s disease mouse brain, in which expression of *MSH3*, but not *DHFR*, correlates with somatic expansion (Tome *et al.*, 2013). Neither *MSH3* nor *DHFR* expression in blood was significantly associated with somatic expansion, onset or progression in this sample. However, investigation in a larger sample, or in a more relevant tissue, such as striatum, would be of interest.

Collectively, our results suggest the *MSH3* 3a repeat allele reduces somatic expansion and improves phenotype in both Huntington’s disease and DM1, potentially through altering *MSH3* expression levels. However, given the proximity of the repeat region to MMR protein binding domains, the 3a allele could also alter MSH3 function in the recognition and repair of insertion-deletion loops, double-strand breaks or single-strand annealing (Lyndaker and Alani, 2009; Schmidt and Pearson, 2016). Repetitive DNA sequences form unusual secondary structures such as slipped strands, hairpin loops, G-quadruplexes and R-loops (Mirkin, 2007; Neil *et al.*, 2017), the stability of which correlates with expansion (Gacy *et al.*, 1995). MSH3 may recognize these structures (Owen *et al.*, 2005) and initiate repair, during which out of register synthesis could result in repeat expansion (Khan *et al.*, 2015; Neil *et al.*, 2017). This preliminary study elucidates variation in *MSH3* that modifies Huntington’s disease and identifies the same signal in an independent trinucleotide repeat disease. Though beyond the scope of the present study, in the future it will be important to replicate these findings in additional independent cohorts for each disease. Together, these results suggest a common mechanism, involving somatic expansion, operates *in vivo* in distinct trinucleotide repeat diseases to influence disease course. Therefore, modulation of MSH3 has significant therapeutic potential in a range of diseases caused by repeat expansions.

## Supplementary Material

awz115_Supplementary_Data

## Abbreviations

DM1: myotonic dystrophy type 1
SNP: single nucleotide polymorphism
TWAS: transcriptome-wide association study

## Appendix 1

### TRACK-HD investigators and OPTIMISTIC consortium

See Supplementary material for full details.

#### TRACK-HD investigators

Peter Kraus, Rainer Hoffman, Alan Tobin, Beth Borowsky, S. Keenan, Kathryn B. Whitlock, Sarah Queller, Colin Campbell, Chiachi Wang, Doug Langbehn, Eric Axelson, Hans Johnson, Tanka Acharya, Dave M. Cash, Chris Frost, Rebecca Jones, Caroline Jurgens, Ellen P. ‘t Hart, Jeroen van der Grond, Marie-Noelle N. Witjes-Ane, Raymund A.C. Roos, Eve M. Dumas, Simon J.A. van den Bogaard, Cheryl Stopford, David Craufurd, Jenny Callaghan, Natalie Arran, Diana D. Rosas, S. Lee, W Monaco, Alison O’Regan, Cassie Milchman, E. Frajman, Izelle Labuschagne, Julie Stout, Melissa Campbell, Sophie C. Andrews, Natalie Bechtel, Ralf Reilmann, Stefan Bohlen, Chris Kennard, Claire Berna, Stephen Hicks, Alexandra Durr, C Pourchot, Eric Bardinet, Kevin Nigaud, Romain Valabrègue, Stephane Lehericy, Cecilia Marelli, Celine Jauffret, Damian Justo, Blair Leavitt, Joji Decolongon, Aaron Sturrock, Alison Coleman, Rachelle Dar Santos, A. Patel, Claire Gibbard, Daisy Whitehead, Ed Wild, Gail Owen, Helen Crawford, Ian Malone, Nayana Lahiri, Nick C. Fox, Nicola Z. Hobbs, Rachael I. Scahill, Roger Ordidge, Tracey Pepple, Joy Read, Miranda J. Say, Bernhard Landwehrmeyer.

#### OPTIMISTIC consortium

Ferroudja Daidj, Guillaume Bassez, Baptiste Lignier, Florence Couppey, Stéphanie Delmas, Jean-François Deux, Karolina Hankiewicz, Celine Dogan, Lisa Minier, Pascale Chevalier, Amira Hamadouche, Michael Catt, Vincent van Hees, Sharon Catt, Ameli Schwalber, Juliane Dittrich, Marie Kierkegaard, Stephan Wenninger, Benedikt Schoser, Angela Schüller, Kristina Stahl, Heike Künzel, Martin Wolff, Anna Jellinek, Cecilia Jimenez Moreno, Grainne Gorman, Hanns Lochmüller, Michael Trenell, Sandra van Laar, Libby Wood, Sophie Cassidy, Jane Newman, Sarah Charman, Renae Steffaneti, Louise Taylor, Allan Brownrigg, Sharon Day, Antonio Atalaia, Joost Raaphorst, Kees Okkersen, Baziel van Engelen, Stephanie Nikolaus, Yvonne Cornelissen, Marlies van Nimwegen, Daphne Maas, Ellen Klerks, Sacha Bouman, Hans Knoop, Linda Heskamp, Arend Heerschap, Ridho Rahmadi, Perry Groot, Tom Heskes, Katarzyna Kapusta, Jeffrey Glennon, Shaghayegh Abghari, Armaz Aschrafi, Geert Poelmans, Shaun Treweek, Fiona Hogarth, Roberta Littleford, Peter Donnan, Adrian Hapca, Michael Hannah, Emma McKenzie, Petra Rauchhaus, Sarah A. Cumming, Darren G. Monckton, Berit Adam, Catharina Faber, Ingemar Merkies.
