# Supplementary material for: MSH3 modifies somatic instability and disease severity in Huntington’s and myotonic dystrophy type 1
Source: Brain. 2019 Jun 19;142(7):1876–86. doi: 10.1093/brain/awz115 (PMC6598626; doi:10.1093/brain/awz115)
Supplement: awz115_Supplementary_Data [file awz115_supplementary_data.zip › awz115-Suppl_data/Supplementary_Data2.pdf]

TRACK-HD

Name	Institution abbreviation	Institution full detail
Peter Kraus	Bochum	University of Bochum, Bochum, Germany
Rainer Hoffman	Bochum	University of Bochum, Bochum, Germany
Alan Tobin	CHDI	CHDI, USA
Beth Borowsky	CHDI	CHDI, USA
S. Keenan	ICL	Imperial College London, London, UK
Kathryn B. Whitlock	Indiana	Indiana University, IN, USA
Sarah Queller	Indiana	Indiana University, IN, USA
Colin Campbell	Indiana/Monash	Indiana University, IN, USA / Monash University, Victoria, Australia
Chiachi Wang	Iowa	University of Iowa, Iowa City, IA, USA
Doug Langbehn	Iowa	University of Iowa, Iowa City, IA, USA
Eric Axelson	Iowa	University of Iowa, Iowa City, IA, USA
Hans Johnson	Iowa	University of Iowa, Iowa City, IA, USA
Tanka Acharya	Iowa	University of Iowa, Iowa City, IA, USA
Dave M. Cash	IXICO	IXICO, London, UK
Chris Frost	LSHTM	London School of Hygiene and Tropical Medicine, London, UK
Rebecca Jones	LSHTM	London School of Hygiene and Tropical Medicine, London, UK
Caroline Jurgens	LUMC, Leiden	Leiden University Medical Centre, Leiden, Netherlands
Ellen P. 't Hart	LUMC, Leiden	Leiden University Medical Centre, Leiden, Netherlands
Jeroen van der Grond	LUMC, Leiden	Leiden University Medical Centre, Leiden, Netherlands
Marie-Noelle N. Witjes-Ane	LUMC, Leiden	Leiden University Medical Centre, Leiden, Netherlands
Raymund A.C. Roos	LUMC, Leiden	Leiden University Medical Centre, Leiden, Netherlands
Eve M. Dumas	LUMC, Leiden	Leiden University Medical Centre, Leiden, Netherlands
Simon J.A. van den Bogaard	LUMC, Leiden	Leiden University Medical Centre, Leiden, Netherlands
Cheryl Stopford	Manchester	St Mary's Hospital, Manchester, UK
David Craufurd	Manchester	St Mary's Hospital, Manchester, UK
Jenny Callaghan	Manchester	St Mary's Hospital, Manchester, UK
Natalie Arran	Manchester	St Mary's Hospital, Manchester, UK
Diana D. Rosas	MGH	Massachusetts General Hospital, Harvard, MA, USA
S. Lee	MGH	Massachusetts General Hospital, Harvard, MA, USA

W Monaco	MGH	Massachusetts General Hospital, Harvard, MA, USA
Alison O'Regan	Monash	Monash University, Victoria, Australia
Cassie Milchman	Monash	Monash University, Victoria, Australia
E. Frajman	Monash	Monash University, Victoria, Australia
Izelle Labuschagne	Monash	Monash University, Victoria, Australia
Julie Stout	Monash	Monash University, Victoria, Australia
Melissa Campbell	Monash	Monash University, Victoria, Australia
Sophie C. Andrews	Monash	Monash University, Victoria, Australia
Natalie Bechtel	Muenster	University of Münster, Münster, Germany
Ralf Reilmann	Muenster	University of Münster, Münster, Germany
Stefan Bohlen	Muenster	University of Münster, Münster, Germany
Chris Kennard	Oxford	University of Oxford, Oxford, UK
Claire Berna	Oxford	University of Oxford, Oxford, UK
Stephen Hicks	Oxford	University of Oxford, Oxford, UK
Alexandra Durr	Paris	APHP, Hôpital Salpêtriere, Paris, France
C Pourchot	Paris	APHP, Hôpital Salpêtriere, Paris, France
Eric Bardinet	Paris	APHP, Hôpital Salpêtriere, Paris, France
Kevin Nigaud	Paris	APHP, Hôpital Salpêtriere, Paris, France
Romain Valabrègue	Paris	APHP, Hôpital Salpêtriere, Paris, France
Stephane Lehericy	Paris	APHP, Hôpital Salpêtriere, Paris, France
Cecilia Marelli	Paris	APHP, Hôpital Salpêtriere, Paris, France
Celine Jauffret	Paris	APHP, Hôpital Salpêtriere, Paris, France
Damian Justo	Paris	APHP, Hôpital Salpêtriere, Paris, France
Blair Leavitt	UBC, Vancouver	University of British Columbia, Vancouver, Canada
Joji Decolongon	UBC, Vancouver	University of British Columbia, Vancouver, Canada
Aaron Sturrock	UBC, Vancouver	University of British Columbia, Vancouver, Canada
Alison Coleman	UBC, Vancouver	University of British Columbia, Vancouver, Canada
Rachelle Dar Santos	UBC, Vancouver	University of British Columbia, Vancouver, Canada
A. Patel	UCL	University College London, London, UK
Claire Gibbard	UCL	University College London, London, UK
Daisy Whitehead	UCL	University College London, London, UK

Ed Wild	UCL	University College London, London, UK
Gail Owen	UCL	University College London, London, UK
Helen Crawford	UCL	University College London, London, UK
Ian Malone	UCL	University College London, London, UK
Nayana Lahiri	UCL	University College London, London, UK
Nick C. Fox	UCL	University College London, London, UK
Nicola Z. Hobbs	UCL	University College London, London, UK
Rachael I. Scahill	UCL	University College London, London, UK
Roger Ordidge	UCL	University College London, London, UK
Tracey Pepple	UCL	University College London, London, UK
Joy Read	UCL	University College London, London, UK
Miranda J. Say	UCL	University College London, London, UK
Bernhard Landwehrmeyer	Ulm	Ulm University, Ulm, Germany

OPTIMISTIC

Name	Location	Role
Ferroudja Daidj	Assistance Publique-Hôpitaux de Paris	Site Investigator
Guillaume Bassez, MD	Assistance Publique-Hôpitaux de Paris	Partner Lead
Baptiste Lignier	Assistance Publique-Hôpitaux de Paris	Site Investigator
Florence Couppey	Assistance Publique-Hôpitaux de Paris	Site Investigator
Stéphanie Delmas	Assistance Publique-Hôpitaux de Paris	Site Investigator
Jean-François Deux, PhD	Assistance Publique-Hôpitaux de Paris	Site Investigator
Karolina Hankiewicz, MD	Assistance Publique-Hôpitaux de Paris	Site Investigator
Celine Dogan	Assistance Publique-Hôpitaux de Paris	Site Investigator
Lisa Minier	Assistance Publique-Hôpitaux de Paris	Site Investigator
Pascale Chevalier	Assistance Publique-Hôpitaux de Paris	Site Investigator
Amira Hamadouche	Assistance Publique-Hôpitaux de Paris	Site Investigator
Michael Catt, PhD	Catt-Sci Ltd.	Partner Lead
Vincent van Hees, PhD	Catt-Sci Ltd.	Site Investigator
Sharon Catt	Catt-Sci Ltd.	Site Investigator
Ameli Schwalber	concentris	Partner Lead
Juliane Dittrich	concentris	Coordinator
Marie Kierkegaard, RPT, PhD	Karolinska Institute Medical University	External Advisory Board Member
Stephan Wenninger, MD	Ludwig-Maximilians-Universität München	Site Investigator
Benedikt Schoser, MD	Ludwig-Maximilians-Universität München	Partner Lead
Angela Schüller, MD	Ludwig-Maximilians-Universität München	Site Investigator
Kristina Stahl	Ludwig-Maximilians-Universität München	Site Investigator
Heike Künzel, MD	Ludwig-Maximilians-Universität München	Site Investigator
Martin Wolff	Ludwig-Maximilians-Universität München	Site Investigator
Anna Jellinek	Ludwig-Maximilians-Universität München	Site Investigator
Cecilia Jimenez Moreno, PhD	Newcastle University	Site Investigator
Grainne Gorman, MD, PhD	Newcastle University	Partner Lead
Hanns Lochmüller, MD, PhD	Newcastle University	Site Investigator
Michael Trenell, PhD	Newcastle University	Site Investigator
Sandra van Laar	Newcastle University	Site Investigator

Libby Wood	Newcastle University	Site Investigator
Sophie Cassidy, PhD	Newcastle University	Site Investigator
Jane Newman, PhD	Newcastle University	Site Investigator
Sarah Charman, PhD	Newcastle University	Site Investigator
Renae Steffaneti, PhD	Newcastle University	Site Investigator
Louise Taylor	Newcastle University	Site Investigator
Allan Brownrigg	Newcastle University	Site Investigator
Sharon Day	Newcastle University	Site Investigator
Antonio Atalaia, MD	Newcastle University	Site Investigator
Joost Raaphorst, MD, PhD	Radboud University	Site Investigator
Kees Okkersen, MD	Radboud University	Site Investigator
Baziel van Engelen, MD, PhD	Radboud University	Chief Investigator
Stephanie Nikolaus	Radboud University	Site Investigator
Yvonne Cornelissen	Radboud University	Site Investigator
Marlies van Nimwegen, PhD	Radboud University	Site Investigator
Daphne Maas	Radboud University	Site Investigator
Ellen Klerks	Radboud University	Site Investigator
Sacha Bouman	Radboud University	Site Investigator
Hans Knoop, PhD	Radboud University	Site Investigator
Linda Heskamp	Radboud University	Site Investigator
Arend Heerschap, PhD	Radboud University	Site Investigator
Ridho Rahmadi	Radboud University	Site Investigator
Perry Groot, PhD	Radboud University	Site Investigator
Tom Heskes, PhD	Radboud University	Site Investigator
Katarzyna Kapusta	Radboud University	Site Investigator
Jeffrey Glennon, PhD	Radboud University	Site Investigator
Shaghayegh Abghari	Radboud University	Site Investigator
Armaz Aschrafi, PhD	Radboud University	Site Investigator
Geert Poelmans, PhD	Radboud University	Site Investigator
Shaun Treweek, PhD	The University of Aberdeen	Partner Lead
Fiona Hogarth, PhD	University of Dundee	Site Investigator

Roberta Littleford, PhD	University of Dundee	Site Investigator
Peter Donnan, PhD	University of Dundee	Partner Lead
Adrian Hapca, PhD	University of Dundee	Site Investigator
Michael Hannah	University of Dundee	Site Investigator
Emma McKenzie	University of Dundee	Site Investigator
Petra Rauchhaus, PhD	University of Dundee	Site Investigator
Sarah A. Cumming, PhD	University of Glasgow	Site Investigator
Darren G. Monckton, PhD	University of Glasgow	Partner Lead
Berit Adam	University of Glasgow	Site Investigator
Catharina Faber, MD, PhD	University of Maastricht	Site Investigator
Ingemar Merkies, MD, PhD	University of Maastricht	Site Investigator
