# Supplementary material for: MSH3 modifies somatic instability and disease severity in Huntington’s and myotonic dystrophy type 1
Source: Brain. 2019 Jun 19;142(7):1876–86. doi: 10.1093/brain/awz115 (PMC6598626; doi:10.1093/brain/awz115)
Supplement: awz115_Supplementary_Data [file awz115_supplementary_data.zip › awz115-Suppl_data/Supplementary_Data.pdf]

Supplementary Fig. 1. Schematic of sequencing design for the *MSH3* exon 1 region. Gene-specific primers with attached MiSeq Illumina barcodes were designed to amplify 534 bp covering the *MSH3* 9 bp tandem repeat region and seven flanking variants. SNP IDs are indicated for each variant, repeat units are coded by coloured boxes, the imputed SNP from GWAS on HD progression is shown in red. Arrows show direction of forward (Read 1) and reverse (Read 2) reads during MiSeq Illumina sequencing, 400 nucleotides were sequenced in a forward read and 200 nucleotides in a reverse read.

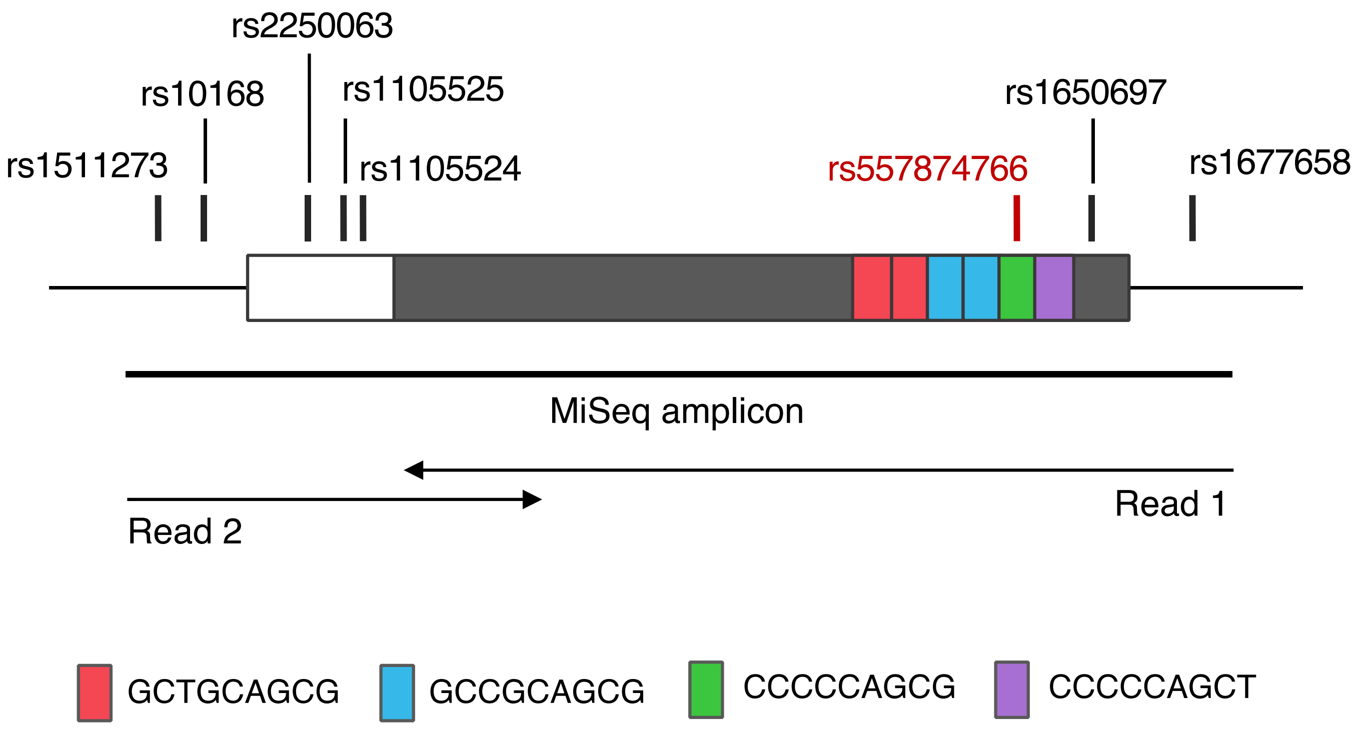

Supplementary Fig. 2. Representative Sanger sequencing of a 3a heterozygote. Two HD samples genotyped as homozygous for the reference allele rs557874766 were subsequently found to have one 3a allele each by MiSeq Illumina sequencing. Sanger sequencing confirmed heterozygosity at the 9 bp tandem repeat, consistent with the MiSeq sequencing result (representative trace shown). On top is the human reference sequence (GRCh38), then the 3a allele sequence (note this is aligns to the reference as separate 27 and 9 bp deletions, light red). The 9 bp tandem repeat is marked in relation to the reference sequence in red.

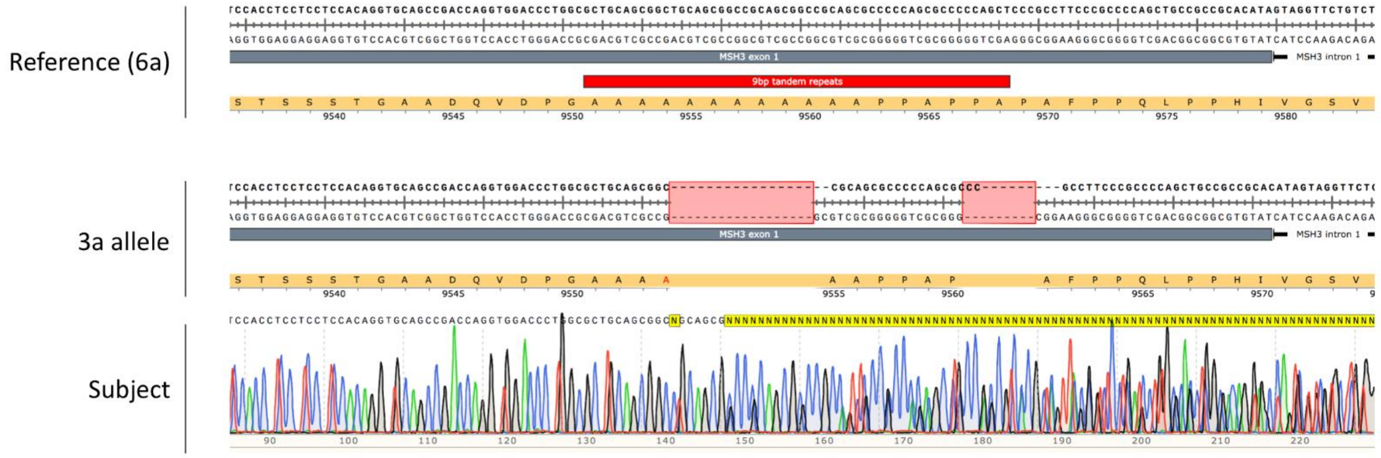

Supplementary Fig. 3. The *MSH3* N-terminal region is poorly conserved between species. Alignment of *MSH3* orthologues of 20 mammals (17 primates). Sequences were obtained from Ensembl and aligned in Clustal. Most mammals have two-repeat alleles (shown in blue and green). Gorillas have the 3a allele, also seen in humans, and chimpanzees have four-repeat allele. In rodents and canines, the 9 bp tandem repeat region is not present.

GRCh38/hg38 chr5:80654861-80654969

```
Human                     accaggtggaccctggcgctgcagcggctgcagcggccgcagcggccgcagcgcccccagcgcccccagctcccgccttcccgccccagctgccgccgcacatagtagg
Chimp                     accaggtggaccctggcgctgcagcggc------------------cgcagcgcccccagcgcccccagcgcccgccttcccgccccagctgccgccgcacgtagtagg
Bonobo                    accaggtggaccctggcgctgcagcggc------------------cgcagcgcccccagcgcccccagcgcccgccttcccgccccagctgccgccgcacgtagtagg
Gorilla                   accaggttgaccctggcgctgcagcggc------------------cgcagcgcccccagcg---------cccgccttcccgccccagctgccgccacacgcagtagg
Orangutan                 accaggtggaccctgacgc---------------------------tgcagcgcccccagcg---------cccactttcccgccccagctgccgccgcacgtagtagg
Gibbon                    accaggtggatcctaacgc---------------------------tgcagcacccccagcg---------cccactgtcccgccccagctgccgctccacgtagtagg
Crab-eating macaque       atcaggtggaccgtgacgc---------------------------cgcagcgcccccagcg---------tccactttcccgccccagctgccgccgctcgtggtagg
Baboon                    atcaggtggaccgtgacgc---------------------------cgcagcgcccccagcg---------tccactttcccgccccagctgccgccgctagtggtagg
Green monkey              atcaggtggaccgtgacgc---------------------------cgcagcgcccccagcg---------tccactttcccgccccagctgccgccgctcgtggtagg
Proboscis monkey          atcaggtggaccatgacgc---------------------------cgcagcgcccccagcg---------tcctctttcccgccccagttgccgccgctcgtggtagg
Golden snub-nosed monkey  atcaggtggaccatgacgc---------------------------cgcagcgcccccagcg---------tccactttcccgccccagttgccgccgctcgtggtagg
Marmoset                  accaggtggaccccgacgc---------------------------cgcagcgcccccagcg---------tccactttcccgccccagctgccgccgcacgtggtggg
Squirrel monkey           accaggtggaccctgacgc---------------------------cgcagcgcccccagcg---------tccactttcccgccccagctgccgccgcacgtggtggg
Tarsier                   gccagacggaccccgactc---------------------------cgcagcgtcccgagcg---------tccgccttcccgct---ggtgcagccacacctggtagg
Bushbaby                  accaggcggactctggctc---------------------------tgcagcgcccccagcg---------tccactttcccgccccaattgccggtacacgtggtggg
Mouse                     agaaggtaaagtacggctc---------------------------cgcggcgcccctagcg---------tccacct--------aggcgaaggccgtcccgggcggg
Dog                       -------------------------------------------------------------------------------------------------------------
Rhesus                    -------------------------------------------------------------------------------------------------------------
Tree shrew                -------------------------------------------------------------------------------------------------------------
Mouse lemur               -------------------------------------------------------------------------------------------------------------
```

Supplementary Fig. 4. MSH3 repeat length correlation with somatic expansion, age at onset, progression score and blood expression of *MSH3* and *DHFR* in HD. Left column – sum of the number of *MSH3* repeats on both alleles, right column – *MSH3* repeat length in repeat homozygotes. Rows in order – residual rate of somatic expansion, age at onset (AAO), progression score, *MSH3* and *DHFR* expression. 79/81 subjects with a sum of 12 repeats are 6 repeat homozygotes.

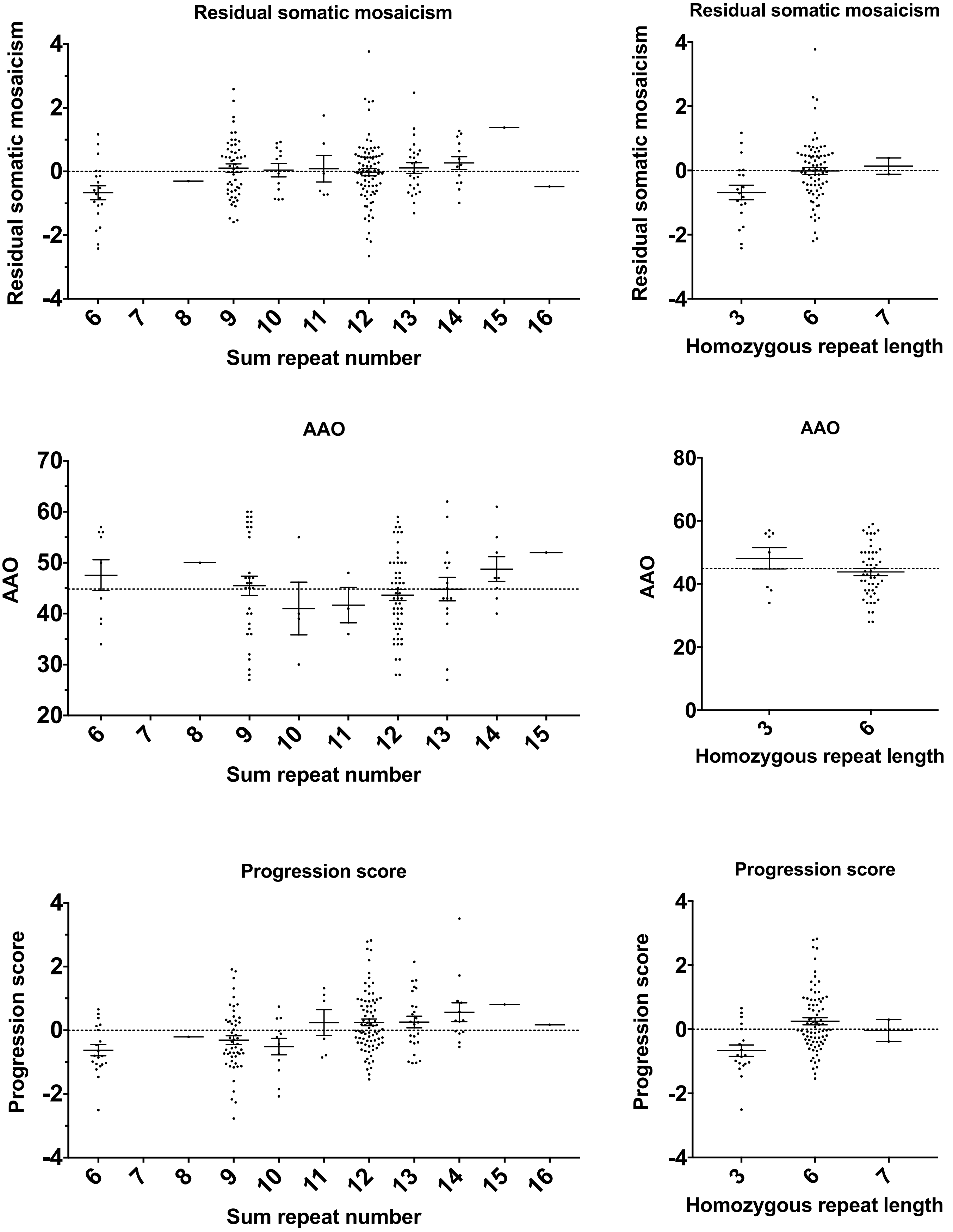

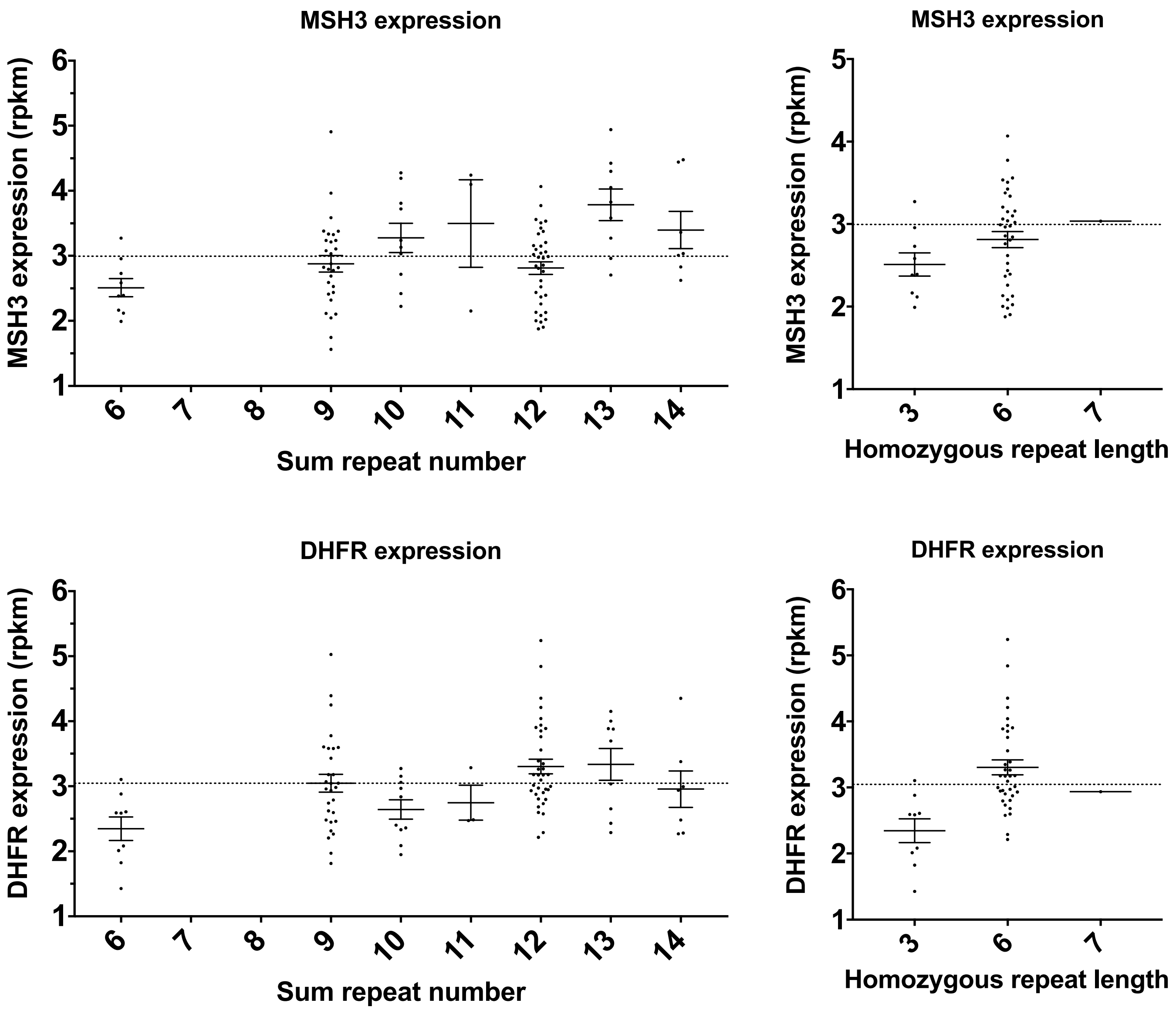
MSH3 expression
MSH3 expression (rpkm)
Sum repeat number
6
7
8
9
10
11
12
13
14
MSH3 expression
MSH3 expression (rpkm)
Homozygous repeat length
3
6
7
DHFR expression
DHFR expression (rpkm)
Sum repeat number
DHFR expression
DHFR expression (rpkm)
Homozygous repeat length
